# The epicaridium larvae of *Paragigantione* species (Isopoda: Epicaridea: Bopyridae) have external yolk sacs: transfer of the genus to Pleurocryptellinae, description of two new species in the genus and a new species of hyperparasite (Isopoda: Epicaridea: Cabiropidae)

**DOI:** 10.1007/s11230-024-10190-z

**Published:** 2024-10-25

**Authors:** Christopher B. Boyko, Jason D. Williams, Gianna Sancetta

**Affiliations:** 1 Department of Biology, Hofstra University, Hempstead, NY 11549 USA; 2 Division of Invertebrate Zoology, American Museum of Natural History, New York, NY 10024 USA

## Abstract

The epicaridean isopods previously known to have epicaridium larvae with posterior yolk sacs were species of *Pleurocryptella* Bonnier, 1900 and a new subfamily, Pleurocryptellinae, was recently erected for this genus. Epicaridium larvae bearing posterior yolk sacs are newly reported from two species of the genus *Paragigantione* Barnard, 1920 which is transferred from Pseudioninae to Pleurocryptelline on the basis of this and other shared characters of adult males and females with species of *Pleurocryptella*. Two new species of *Paragigantione* are described, one from the northeast Atlantic based on type material that was misidentified as belonging to the type species of the genus, *P*. *papillosa* Barnard, 1920 and a second from off New Zealand. One specimen of the New Zealand species had a cryptoniscus larva of a new species of hyperparasite of the genus *Bourdonia* Rybakov, 1990 in the marsupium while another had a species of *Duplorbis* (Rhizocephala); the former is described based on this material as well as a specimen from a specimen of *Pseudione* cf. *fibriata* Richardson, 1910 from New Zealand. Keys to species of *Paragigantione* for both males and females are provided.

## Introduction

Parasitic isopods of the family Bopyridae (currently 648 species; Boyko et al., 2024) infest a range of crustaceans as definitive hosts including a diverse array of squat lobsters (Boyko et al., 2012). Recently, the aberrant genus *Pleurocryptella* Bonnier, 1900 was placed in its own subfamily, Pleurocryptellinae, due to females having oostegites on the sixth and seventh pereomeres, anterior and posterior lobes of the first oostegites rounded, males with segmented maxillipeds, well-developed pleopods and articulated uropods, and epicaridium larvae having posterior yolk sacs (Williams et al., 2024), many of which have historically been interpreted as primitive characters (e.g., Shiino, 1952, 1965; Markham, 1986). No single one of these characters was unique to *Pleurocryptella* except the presence of posterior yolk sacs on the larvae.

Examination of New Zealand and Florida specimens of species belonging to the genus *Paragigantione* Barnard, 1920 revealed epicaridium larvae that all also possess posterior yolk sacs. All species of *Paragigantione* also share with those of *Pleurocryptella* having the anterior and posterior lobes of the first oostegites rounded and males with segmented maxillipeds, well-developed pleopods and articulated uropods. However, females of species of *Paragigantione* do not have oostegites on the sixth and seventh pereomeres and the diagnosis of Pleurocryptellinae must be modified to accommodate this character state variability. Based on adult and larval characters, *Paragigantione* is therefore transferred from Pseudioninae to Pleurocryptellinae.

In the present paper we report on the adult (female and male) and larval (epicaridium) morphology of a new species of *Paragigantione* found parasitizing the squat lobster *Gonionida rubrimana* (Ahyong) as well as an unidentified munidid. We also describe the epicaridium larvae of *Paragigantione americana* (Markham, 1974b) for the first time and identify specimens of *Paragigantione papillosa* Barnard, 1920 *sensu* Bourdon (1981) as a new species from the eastern Atlantic. Keys to species of *Paragigantione* for both males and females are provided. In addition, one female specimen of the new *Paragigantione* species from New Zealand contained a hyperparasitic isopod which is described herein as a new species of *Bourdonia* Rybakov, 1990 based on the cryptoniscus larval stage; a second specimen of this hyperparasite was found infesting a female *Pseudione* cf. *fibriata* Richardson, 1910. This is the first description of a hyperparasitic isopod from any species of *Paragigantione*.

## Material and methods

Carapace lengths (CL) of hosts were measured using calipers. Parasite sizes are given as maximal total length (TL). All measurements were made with an ocular micrometer, from drawing tube sketches, or from scale bars in SEM images.

Original line drawings were made by using drawing tubes attached to Olympus compound (Olympus CX41) and dissecting microscopes (Olympus SZX12). Adobe Illustrator and a Wacom Cintiq pen display was used to trace original sketches and produce final figures. Light micrographs were created with a Macropod Pro kit (MacroscopicSolutions) and resulting pictures were aligned and stacked with the focus stacking software Zerene Stacker (10–20 images from bottom to top of specimens).

For Scanning Electron Microscopy (SEM) preparation of epicaridium and cryptoniscus larvae, specimens were dehydrated in an ascending ethanol (EtOH) series ending with 100% EtOH. Specimens were then dried in a Samdri 795 Critical Point Dryer (Tousimis, Rockville, MD, USA), mounted on aluminum stubs, coated with gold using an EMS-550 Sputter coater (Electron Microscopy Sciences, Hatfield, PA, USA), and viewed with a FEI Quanta 250 SEM (Thermo Fisher Scientific, Waltham, MA, USA).

Specimens are deposited in the Muséum National d’Histoire Naturelle, Paris (MNHN), Voss Marine Invertebrate Collections, Rosenstiel School of Marine and Atmospheric Science, University of Miami (UMML), and the National Institute of Water & Atmospheric Research Ltd (NIWA), Wellington, New Zealand. References are provided for taxonomic authorities of parasite taxa but not for those of hosts.

## Systematics

Order Isopoda Latreille, 1816

Suborder Epicaridea Latreille, 1825

Superfamily Bopyroidea Rafinesque, 1815

Family Bopyridae Rafinesque, 1815

Subfamily Pleurocryptellinae Williams & Boyko *in* Williams, Boyko & Stewart, 2024

Type genus.— *Pleurocryptella* Bonnier, 1900.

Other included genus.— *Paragigantione* Barnard, 1920

Emended diagnosis.– **Female **ovate-elongate, body distorted; head bilobed or not bilobed; frontal lamina present. Eyes absent. Maxilliped without palp or with setose articulated and segmented palp. Barbula with two long smooth lobes or one long lobe and one small nub or one long smooth lobe on each side, median region smooth. Five or seven pairs of oostegites; oostegite 1 with ovate posterior lobe, smaller, subequal or larger than anterior lobe; internal ridge smooth. Coxal plates, dorsolateral bosses and tergal projections present. Mediodorsal lobes absent. Pereopods not elongate; without propodal sockets. Pleon not narrower than pereon. Lateral plates absent or reduced; five pairs of smooth biramous pleopods or four pairs of smooth biramous pleopods and one pair of smooth uniramous pleopods; uropods uniramous, smooth, distally entire or bilobed. **Male** approximately two to four times as long as wide, head narrower than or as wide as pereon, pereomeres not narrower posteriorly. Eyes absent. Maxillipeds segmented. Pereopods 1 and 2 larger than or subequal to other pairs. Midventral tubercles present. Pleon of six pleomeres; five pairs of pleopods; posterolateral corners of pleomere 6 rounded or indented; articulated uropods present. **Epicaridium larva** body tear-drop shape with posterior yolk sac, extending between uropods.

Genus *Paragigantione* Barnard, 1920

Type species.— *Paragigantione papillosa* Barnard, 1920, by monotypy.

Other included species.— *Paragigantione indica* (Nierstrasz & Brender à Brandis, 1923), *Paragigantione americana* (Markham, 1974b), *Paragigantione europaea* **n. sp.**, *Paragigantione sadieae* **n. sp.**

Remarks.– *Paragigantione* and the type species, *P*. *papillosa*, were incompletely described and figured by Barnard (1920) but the syntype female was redescribed and figured by Bourdon (1972a), who later (Bourdon, 1981) concluded that *Bonnieria* Nierstrasz & Brender à Brandis, 1923 was a synonym of *Paragigantione*. However, Bourdon (1981) erred in identifying his new specimens from the Celtic Sea as conspecific with the syntype female from South Africa; the 1981 material is described as a new species herein.

**Fig. 1:**
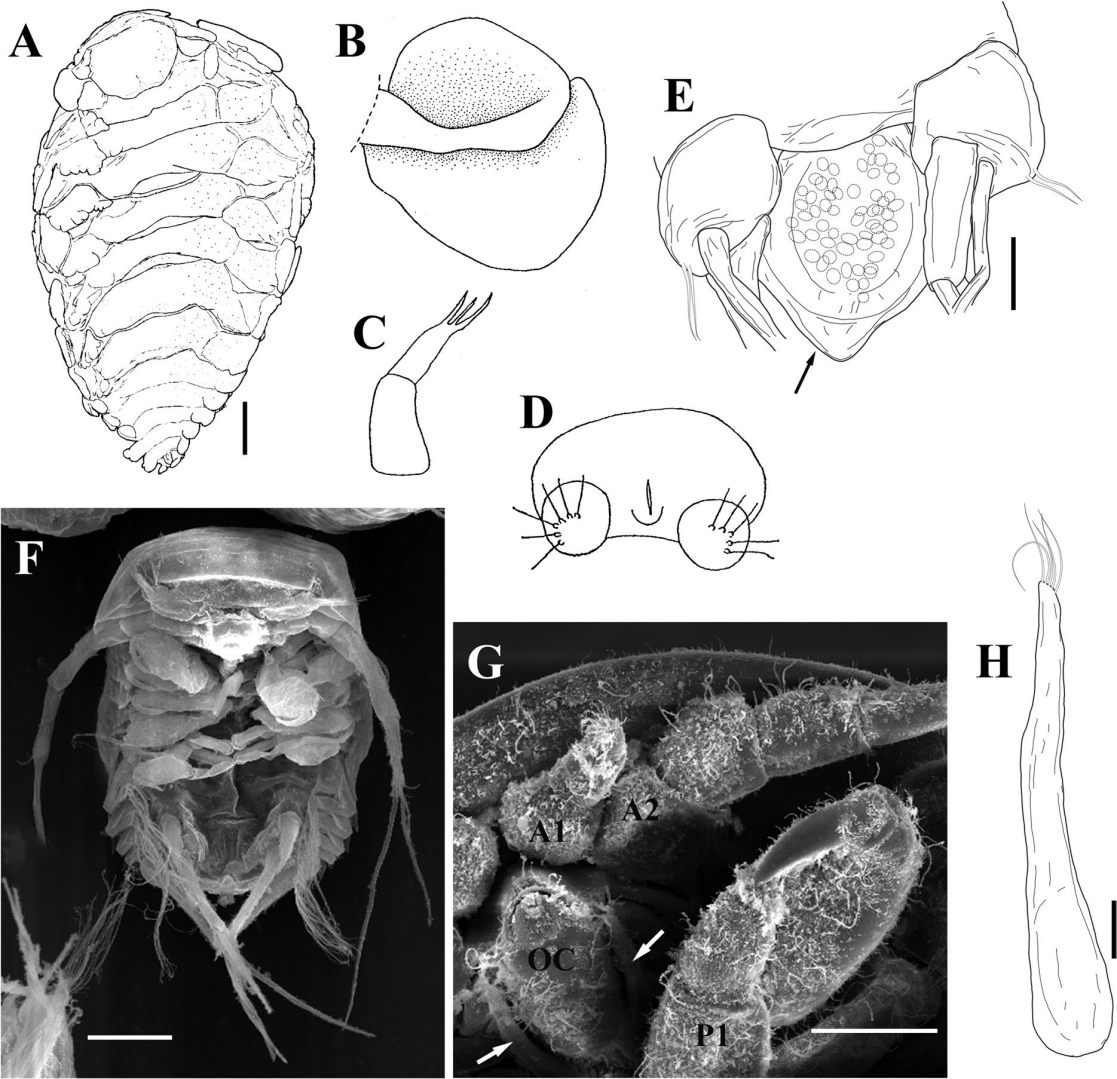
*Paragigantione americana* (Markham, 1974b) (**A, E**), *Paragigantione papillosa* Barnard, 1920 (**B–D**), and *Orthione griffenis* Markham, 2004 (**F–H**). **A**, Female dorsal view; **B**, Oostegite 1, inner view; **C**, Maxilliped of male; **D**, Pleotelson of male showing articulated and setose uropods; **E**, Epicaridium larva, posterior end showing yolk sac (arrow) between uropods; **F**, Epicaridium larva, ventral view; **G**, Anterior end of male, ventral view showing maxillipeds (arrows), antennule (A1), antenna (A2), oral cone (OC), and pereopod 1 (P1); **H**, Maxilliped of male. *Scale-bars*: **A**, 1 mm, **E**, **H**, 100 µm, **F**, 50 µm, **G**, 250 µm; rest not to scale. **A** modified from Markham (1974b); **B–D** modified from Bourdon (1981).

*Paragigantione* is distinctive in that females of all species have the autapomorphy of distally bifurcated uniramous uropods (Figs. 1A, 3A, H, 6A, J) while also possessing rounded anterior and posterior lobes of the first oostegite (Figs. 1B, 3D, 6G, H); males have segmented maxillipeds (Figs. 1C, 4C, 7E) and articulated uropods (Fig. 1B, 4E, 7G). The rounded posterior lobe of the first oostegite of females and segmented maxillipeds and articulated uropods of males are, as has been long known, shared with all species of *Pleurocryptella* Bonnier, 1900. The discovery of a new synapomorphy with *Pleurocryptella*, the presence of large posterior yolk sacs on the epicaridium larvae (Fig. 8A, F), indicates that *Paragigantione* belongs to the same subfamily as *Pleurocryptella* and we herein transfer it to Pleurocryptellinae. This action is taken although females of *Paragigantione* spp. have only five pairs of oostegites, in contrast to the seven pairs in *Pleurocryptella* spp. but as seven pairs of oostegites is not an autapomorphy for *Pleurocryptella* (see Williams et al., 2024), we give greater weight to the larval characters as well as the presence of segmented maxillipeds in males. There are other genera, e.g., *Gigantione* Kossmann, 1881, *Parapleurocryptella* Bourdon, 1972b, and *Pagurocryptella* Boyko & Williams, 2010, with species where adults have some of these character states but their epicaridium larvae are unknown. Species of *Orthione* Markham, 2004 also have some similarities to those of *Pleurocryptella* but molecular data (Williams et al., 2024) does not place them in the same clade, the epicaridium larvae lack posterior yolk sacs (Fig. 1F) and males have unsegmented maxillipeds (Fig. 1G, H).

To date, species of *Paragigantione* have been found on the following hosts: *P*. *papillosa* from *Munida sanctipauli* Henderson (a misidentification, actually *Gonionida benguela* (de Saint Laurent & Macpherson); see Baba et al., 2008)) from South Africa (1700-1810 m), *P*. *indica* Nierstrasz & Brender à Brandis, 1923 from *Munida militaris* var. *curvirostris* Henderson (now *Gonionida curvirostris* (Henderson, 1885); a possible misidentification and may be *Gonionida militaris* (Henderson); see Baba et al., 2008) from off Indonesia (538 m), and *P*. *americana* from *Munida microphthalma* A. Milne-Edwards (now *Typhlonida microphthalma* A. Milne-Edwards)) off Guyana (1220-1140 m). The material from Bourdon (1981) is described herein as *P*. *europaea* **n. sp.** from *Munida sanctipauli* (= *Typhlonida sanctipauli*).

**Fig. 2:**
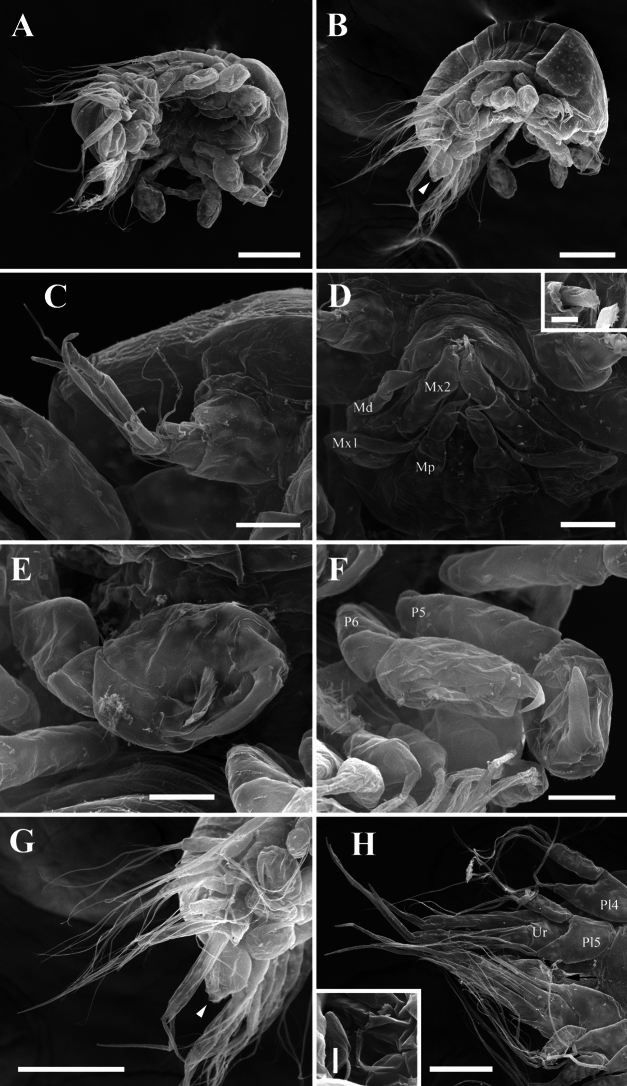
Epicaridium larvae of *Paragigantione americana* (Markham, 1974b). **A**, Ventral view; **B**, Lateral view, yolk sac shown by arrowhead; **C**, Left antennule; **D**, Mouthparts (Md = mandibles, Mp = maxilliped, Mx1 = maxillula, Mx2 = maxilla), inset shows tips of mandibles;** E**, Pereopod 1;** F**, Pereopods 5, 6 (P5, P6);** G**, Lateral view of pleopods, uropods and yolk sac shown by arrowhead;** H**, Ventral view of posterior pleopods 4, 5 (PL4, PL5), uropods (Ur), and yolk sac, base of yolk sac with opening shown by arrow, inset shows close-up of opening. *Scale-bars*: **A, B, G, H**, 100 µm, **C, E**, 20 µm, **D, F**, 25 µm, **D** inset, 5 µm, **H** inset, 2.5 µm

***Paragigantione americana*** **(Markham, 1974b)** (Figs. 1E, 2)

*Bonnieria americana* Markham, 1974a: 40–48, figs. 6–9 (unpublished dissertation, unavailable name).—Markham, 1974b: 614–620, fig. 1–4 (08°40′N, 57°38′W, off Guyana, 1220–1440 m).—Wenner & Windsor, 1979: 295, 302 (Norfolk Canyon, ex *Typhlonida microphthalma* (A. Milne-Edwards)).—Wenner, 1982: 365 (Mid-Atlantic Bight, ex *T*. *microphthalma*).

*Paragigantione americana*.—Bourdon, 1981: 623–624 (reassignment to *Paragigantione*).—Markham, 1988: 56 (list).—Brito et al., 2018: 444 (mention).—Cardoso, 2010: 62–63 (off Rio de Janeiro, Bacia de Campos, Brazil, 1105–1605 m, ex *T*. *microphthalma*).—Markham, 2020: 146 (list).—Ribeiro & Horch, 2023: 163 (list).

Material examined.– “Larvae of *Bonnieria* sp #1 from *Munida microphth*.” = cf. 50 epicaridium larvae on slide from female holotype (8.6 mm, USNM 141592) ex *Munida microphthalma* A. Milne-Edwards (= *Typhlonida microphthalma* (A. Milne-Edwards)), B. S. Mayo, det. of host, Pillsbury Sta. P-689, off coast of Guyana, 08°40′N, 57°38′W, 1220-1440 m, 15 July 1968 (UMML); additional larvae extracted from holotype.

Type locality.– 08°40′N, 57°38′W, off Guyana, 1220–1440 m.

Distribution.– Mid-Atlantic Bight to Bacia de Campos, Brazil, 750–1698 m.

Host.– *Typhlonida microphthalma* (A. Milne-Edwards).

Description of mature epicaridium larva (Figs. 1E, 2).– Approximately 565 µm in length when extended (anterior margin of head to end of yolk sac). Body broadest at anterior end, tapering distally (Fig. 2A, B). Anterior margin of head rounded. Antennules of three articles each (Fig. 2C), basal article rounded, with anterodistal seta, branched at tip; article 2 quadrate with approximately four setae surrounding base of article 3, some with branched tips; article 3 complex, small basal portion with one short and one longer digitiform lobe each bearing two curled terminal setae with setules, three aesthetascs with clear divisions along their length (Fig. 2C). Antenna and terminal setae approximately as long as entire body (Fig. 2A, B); antenna of six articles (four basal and two flagellar), articles 1 and 2 subequal in size; articles 3 and 4 progressively longer, flagellar article 1 approximately same length as terminal basal article but markedly thinner, flagellar article 2 shorter and with two short terminal setae and two long setae, exceeding length of flagellar articles.

Mouthparts: stout mandibles distally toothed (Fig. 2D and inset), maxillula (= maxilla 1) below mandibles, becoming thin toward distal end and slightly coiled along length; mandibles and maxillules surrounded by maxilla (= maxilla 2); maxillipeds of two articles each, basal article broad, triangular, distal article elongate and bearing one medial stout seta and two distal stout setae (Fig. 2D).

Six pairs of gnathopodal pereopods, subequal in size, each with curved dactylus, tip of dactylus extending to anterior portion of carpus, carpus fused with rounded propodus, two large multifid scales on propodus, closely applied to side of dactylus, merus triangular, with terminal seta, ischium ovate, basis elongated (Fig. 2E, F); spinous scales or spinular combs most prominent on propodus (Fig. 2E, F).

Pleon with five pairs of biramous pleopods (Fig. 2G, H); coxopodite and endopodite of each fused, endopodite with two long, plumose terminal setae, exopodite with three long, plumose terminal setae. Uropods biramous, cylindrical peduncle stout with two small lateral setae, endopod slightly longer than exopod, endopods and exopods with two stout terminal setae each, medial seta longer than outer seta (Fig. 2G, H). Yolk sac between uropods, extending approximately half length of uropods, distal margin rounded (Figs. 1E, 2A, B, G, H), small opening on ventral side of yolk sac peduncle (Fig. 2H inset); yolk sac with lipid content within central portion (Fig. 1E).

Remarks – The adult male and female of *P*. *americana* were well-described by Markham (1974b). Although he made a slide preparation (now in UMML) of epicaridium larvae obtained from the holotype, he did not mention anything about them in either his dissertation (Markham, 1974a) or in the published species description (Markham, 1974b). This is the first description of the epicaridium larvae of this species, based on both the slide preparation of Markham and larvae newly extracted from the holotype, and clearly shows the presence of yolk sacs on all larval specimens. As reported by Williams et al. (2024) for at least some species of *Pleurocryptella*, the epicaridium larvae of *P. americana* exhibit a small opening toward the base of the yolk sac peduncle. Presumably, the lining of the yolk sac is contiguous with the digestive tract and this opening at the base of the penduncle is the anus, but histological studies are needed to clarify the connections and other modifications of the digestive system in these larvae.

**Fig. 3:**
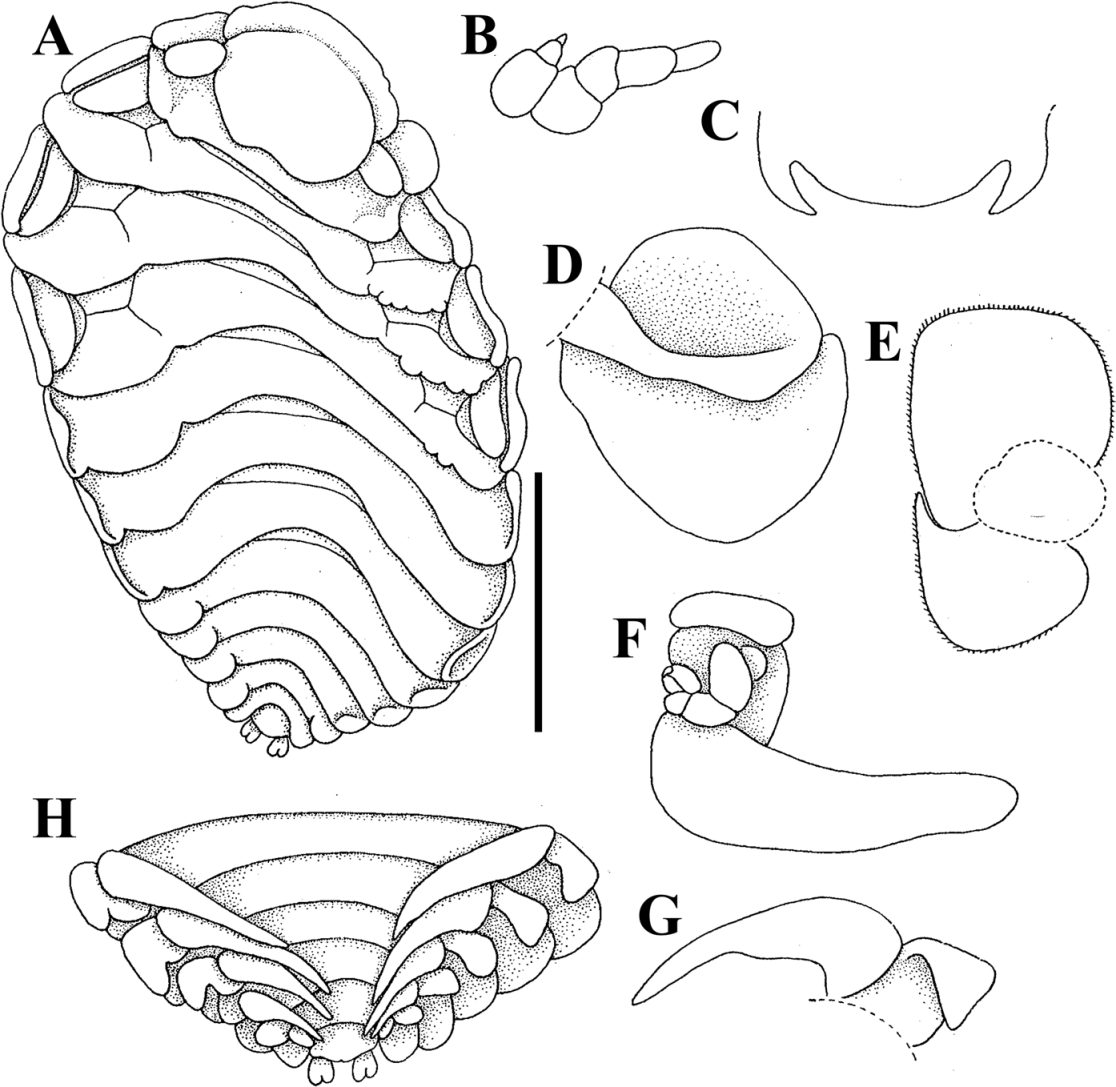
Female of *Paragigantione europaea* **n. sp.** **A**, Dorsal view; **B**, Left antennae; **C**, Barbula; **D**, Left oostegite 1, inner view; **E**, Left maxilliped; **F**, Right oostegite 5 and pereopod 5; **G**, Left pleopod 1; **H**, Pleon, ventral view. *Scale-bars*: **A**, 2 mm, rest not to scale. **A–H** modified from Bourdon (1981).

**Fig. 4:**
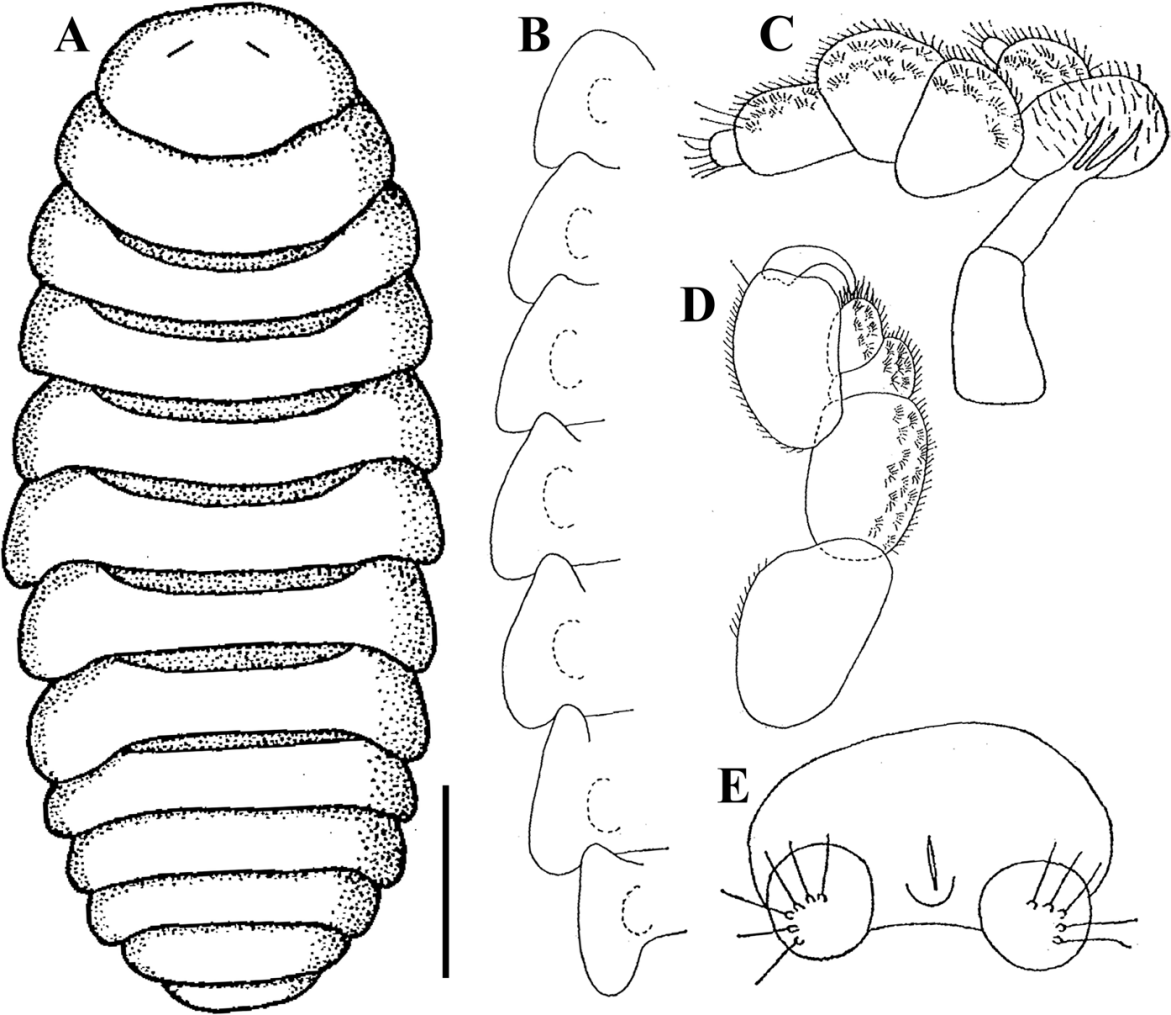
Male of *Paragigantione europaea* **n. sp.** **A**, Dorsal view; **B**, Pereomeres, right side, ventral view; **C**, Right antenna, antennule, and maxilliped; **D**, Right pereopod 1; **E**, Pleon and uropods. *Scale-bars*: **A**, 0.5 mm, rest not to scale. **A–E** modified from Bourdon (1981).

***Paragiantione europaea*** **n. sp.** (Figs. 3, 4)

*Paragigantione papillosa*.— Bourdon, 1981: 618–624, figs. 2–4 (not *Paragigantione papillosa* Barnard, 1920).

Material examined.– None; the holotype female and allotype male are MNHN Ep. 88 (*fide* Bourdon, 1981).

Type locality.– Thalassa Sta. Z 454, Whittard Canyon, 48°37’01”N, 10°53’04”W, Celtic Sea, 1700–1810 m, 28 Oct 1973, coll. CENTOB (Centre National de tri d’Océanographie Biologique).

Distribution.– Known only from the type locality.

Host.– *Typhlonida sanctipauli (*Henderson).

Description.– Holotype female: body length 5.8 mm, maximal width 3.9 mm across pereomeres 3 and 4, head length 1.1 mm, head width 1.4 mm. Body sinuous, ovate, pereon slightly deflexed sinistrally; all body regions and pereomeres distinctly segmented (Fig. 3A). Head ovate, broader than long, bilobed on posterior margin, frontal lamina broad; eyes absent (Fig. 3A). Barbula with one smooth digitiform lobe on each side, evenly rounded median region (Fig. 3C). Antennules of three articles each, terminal article minute; antennae of four articles each, terminal article approximately half as wide as penultimate article, setose (Fig. 3B; setae not shown in figure). Maxilliped with setae on margin of anterior lobe, palp absent; posterior lobe with setose short subacute spur (Fig. 3E). Oostegite 1 anterior lobe ovate, posterior lobe approximately two times as larger than anterior lobe, internal ridge smooth (Fig. 3D). Oostegite 5 without fringe of setae (Fig. 3F).

Pereon of seven pereomeres, broadest across pereomeres 3 and 4, gradually tapering anteriorly and posteriorly; pereomere 1 with straight posterior margin; pereomere 2 anteriorly straight, posteriorly weakly concave, pereomeres 3–7 anteriorly convex, posteriorly concave (Fig. 3A). Coxal plates distinct on pereomeres 1–4, indistinct and recurved on 5–7, dorsolateral bosses on pereomeres 1–4, absent on pereomeres 5–7; tergal projections on pereomeres 2–4 on short side of body with posterior crenulation (Fig. 3A). Marsupium open. Pereopods subequal, bases without bosses (Fig. 3F).

Pleon with six pleomeres including pleotelson; pleomeres 1–5 with rounded lateral plates (Fig. 3A). Pleomeres 1–5 with biramous pleopods, decreasing in size posteriorly, not covering median region of pleon; endopods of 1–4 lanceolate, longer than exopods, pleopod 5 with endopod and exopod rounded, endopod shorter (Fig. 3G, H); uropods uniramous, each with distal bifurcation, anal cone absent (Fig. 3H).

Allotype male: length 2.6 mm, maximal width 1.1 mm across pereomere 5, head length 0.31 mm, head width 0.52 mm, pleon length 0.7 mm. All body segments with numerous setose scales, most numerous on lateral edges (Fig. 4A). Head ovoid, widest at posterior margin, distinct from pereomere 1, medial margin slightly extending into pereomere 1; sensory pits present, eyes absent (Fig. 4A). Antennules of three articles each, all articles setose; antennae of four articles each, all articles setose (Fig. 4C). Maxilliped two-segmented with three or four terminal thick setae (Fig. 4C).

Pereomere 5 broadest, others tapering slightly anteriorly and posteriorly (Fig.4A). Pereomeres 1–5 weakly concave, pereomeres 6 and 7 straight, distolateral margins of all pereomeres rounded (Fig 4A); ventral anterolateral edges of pereomeres 2–7 forming extended apophysis, larger on posterior pereomeres (Fig. 4B). Pronounced midventral tubercle on pereomere 1, wider but weakly demarcated midventral tubercles on pereomeres 2–7. Pereopods 1 and 2 each with long, curved dactylus reaching to carpus (Fig. 4D), posterior dactyli decreasing in size; propodus large, broad, fringed with setae on dorsal edge; carpus and merus small, rounded with numerous setose squamae on ventral half and fringe of setae on ventral margin; ischium and basis rounded, ischia with numerous setose squamae on ventral half and fringe of setae; bases with fringe of setae in median of dorsal margin (Fig. 4D).

Pleon of six pleomeres, sides of all pereomeres curled ventrally and overlapping (Fig. 4A); small, rounded pleopods on pleomeres 1–5. Pleotelson curled ventrally, not visible in dorsal view, with articulated, rounded uropods bearing four to six distal setae and extending beyond anal cone (Fig. 4E).

Remarks.— Bourdon (1972a) redescribed *Paragigantione papillosa* based on the female syntype of Barnard (1920), the male syntype not being mentioned and possibly lost, and later reported on purported new material of the species (Bourdon, 1981). Comparison of the descriptions and illustrations from Bourdon’s two publications of what is supposedly the same species reveals several key differences between them. For females, the syntype of *P*. *papillosa* has only coxal plates present whereas the female of Bourdon (1981; fig. 3A herein) has coxal plates, dorsolateral bosses, and tergal projections with the anterior tergal projections on the short side of the body being crenulate on the posterior margins, the uropods of the syntype are much more deeply bifurcated than those of Bourdon’s (1981; fig. 3A, H herein) specimen, and the posterior lobe of the first oostegite is longer than the anterior lobe and distally narrowing whereas that of Bourdon’s (1981, fig. 3D herein) specimen is subequal in length to the anterior lobe and broad distally. A comparison with the male syntype described and figured by Barnard (1920) with that of Bourdon (1981) shows that the pleotelson of the male is visible in dorsal view in the syntype but not visible in the male of Bourdon (1981, fig. 4A herein) and there are midventral tubercles on pereomeres 1–6 of the syntype but on 1–7 of Bourdon’s (1981) male. Epicaridium larvae of *P*. *papillosa* and Bourdon’s (1981) specimens are unknown.

The pair of specimens described and illustrated by Bourdon (1981) is not conspecific with *P*. *papillosa* but are a distinct species, here named *P*. *europaea* **n. sp.**, that appears much more closely related to *P*. *americana* than to *P*. *papillosa*. *Paragigantione europaea* **n. sp.** and *P*. *americana* share the following character states: (1) females with anterior bosses on the short side of the body being crenulate on the posterior margins, (2) posterior lobe of oostegite 1 subequal in length to the anterior lobe and broad distally, (3) uropods of the female moderately bifurcated, (4) males with pleotelson not visible in dorsal view, and (5) males with midventral tubercles on pereomeres 1–7. *Paragigantione europaea* **n. sp.** can be distinguished from *P*. *americana*, its putative sister species, by the proportions of the pleopods (pleopodal endopod 1.3x as long as exopod in *P*. *americana*; 3.5x as long in *P*. *europaea* **n. sp.**) and presence of midventral tubercles on pleomeres of the male (present on pleomeres 1 and 2 in *P*. *americana*; absent on any pleomeres in *P*. *europaea* **n. sp.**).

It might be asked why Bourdon considered the types of *P*. *papillosa* and his 1981 specimens as conspecific, especially as the species was originally described from South Africa but the 1981 specimens were collected in the northeast Atlantic in the Celtic Sea. He may have been influenced by the identification of the host of the 1981 material supposedly being the same as that of the type specimens: *Munida sanctipauli* (= *Typhlonida sanctipauli* (Henderson)). However, *T*. *sanctipauli* does not occur in South Africa (see Baba et al., 2008) and that host was almost certainly *Gonionida benguela* (de Saint Laurent & Macpherson). The host for the types of *P*. *europaea* **n. sp.** was likely correctly identified as *T*. *sanctipauli*.

**Fig. 5:**
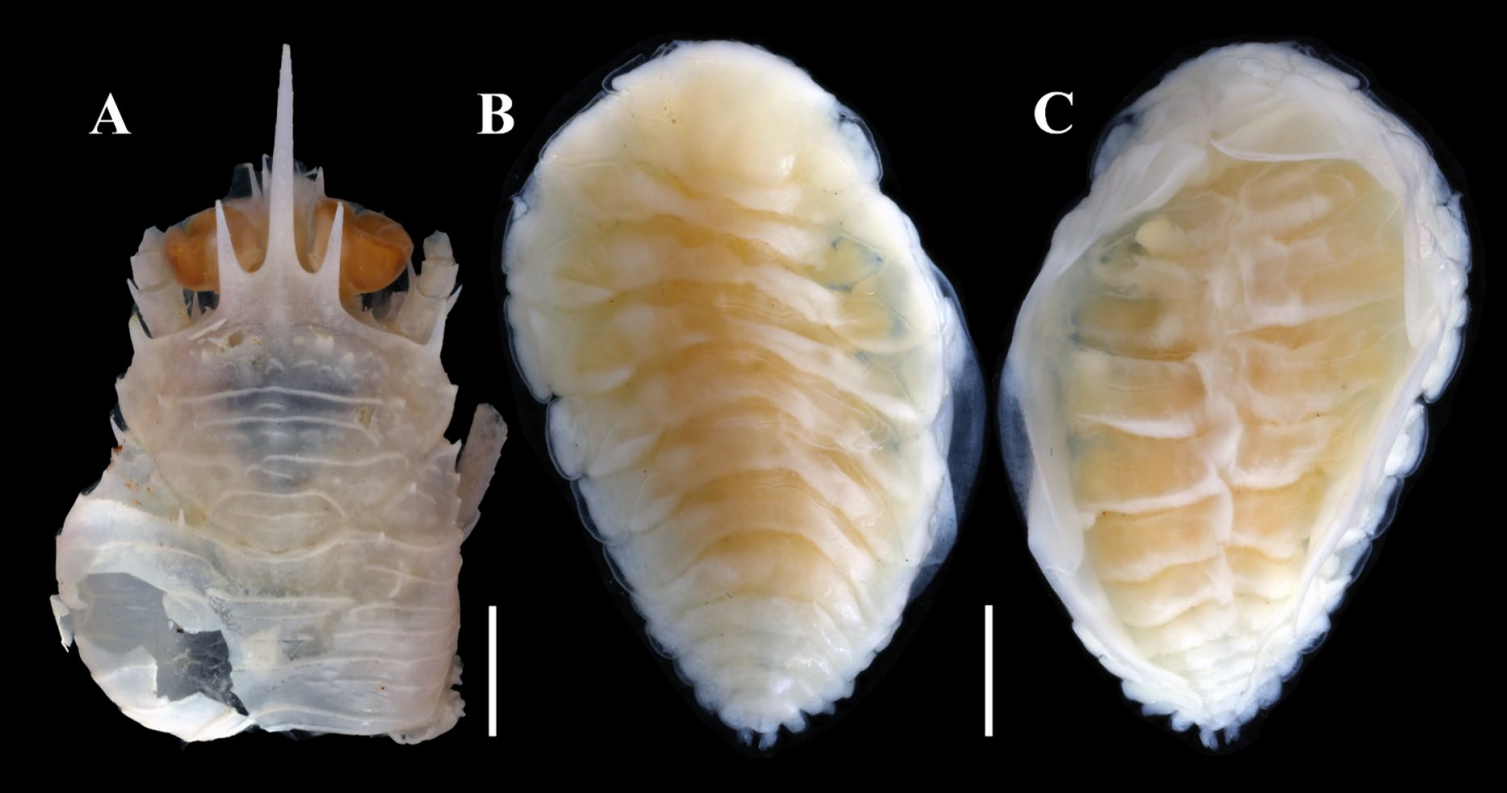
Squat lobster host *Gonionida rubrimana* (**A**) parasitized by *Paragigantione sadieae* **n. sp.** (**B, C**). **A**, Dorsal view of host *G. rubrimana* showing left branchial chamber that contained parasite; **B**, Dorsal view of holotype female of *P*. *sadieae* **n. sp.**; **C**, Ventral view of holotype female *P*. *sadieae* **n. sp.** *Scale-bars*: **A**, 2.5 mm, **B, C**, 1 mm.

**Fig. 6:**
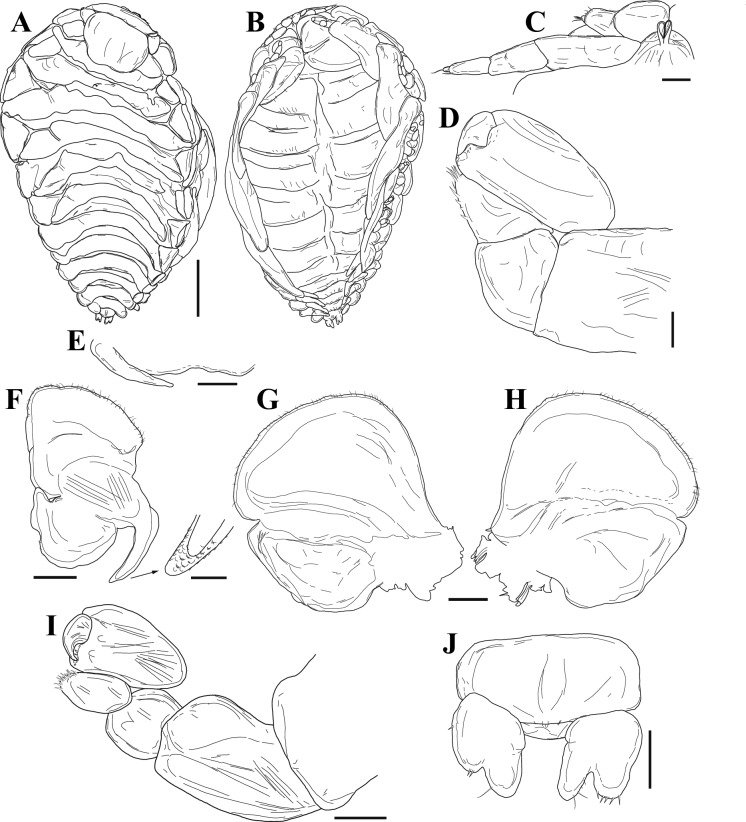
Holotype female of *Paragigantione sadieae* **n. sp.** **A**, Dorsal view; **B**, Ventral view; **C**, Right antennae; **D**, Left pereopod 7; **E**, Middle and left side of barbula; **F**, Left maxilliped (inset = tip of barbula lobe); **G**, Left first oostegite, outer view; **H**, Left first oostegite, inner view; **I**, Left pereopod 1; **J**, Pleon and bifurcated uropods. *Scale-bars*: **A, B**, 1 mm, **C**, **I**, 100 µm, **D, F** (inset), **J**, 50 µm, **E–H**, 250 µm.

**Fig. 7:**
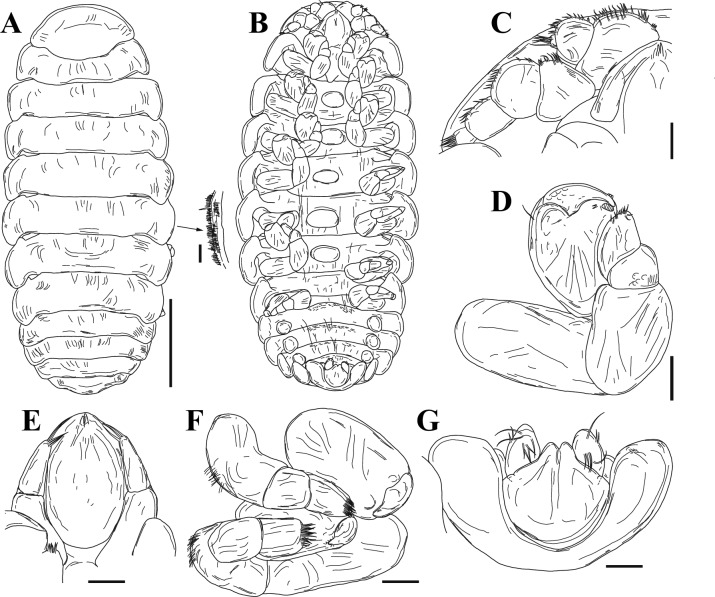
Allotype male of *Paragigantione sadieae* **n. sp.** **A**, Dorsal view (inset of left side of segment 5 showing setae); **B**, Ventral view; **C**, Right antenna and antennule; **D**, Right pereopod 1; **E**; Oral cone and maxillipeds; **F**, Left pereopods 6, 7; **G**, Pleon and uropods. *Scale-bars*: **A, B**, 0.5 mm, **A** (inset), 5 µm, **C–G**, 50 µm.

**Fig. 8:**
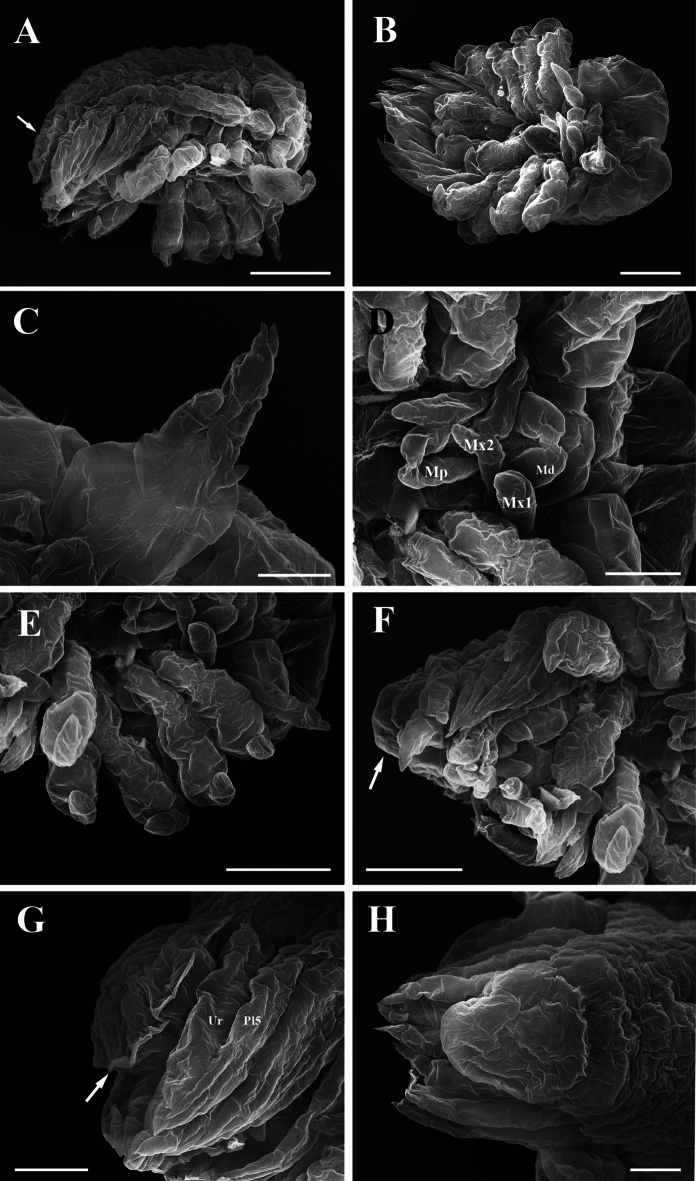
Epicaridium larva of *Paragigantione sadieae* **n. sp.** **A**, Lateral view; **B**, Ventral view with antenna and antennules; **C**, Right antennule; **D**, Mouthparts (Md = mandibles, Mp = maxilliped, Mx1 = maxillula, Mx2 = maxilla); **E**, Pereopods 1–5; **F**, Pleon, dorsal view; **G**, Lateral view of yolk sac, uropod (Ur), and pleopod 5 (Pl5); **H**, Dorsal view of yolk sac. Arrows indicate dorsal yolk sac (**A, F**) and yolk sac ridge (**G**). *Scale-bars*: **A, B, E, F**, 50 µm,** C**, 15 µm, **D, G, H**, 25 µm

***Paragigantione sadieae*** **n. sp.** Figs. 5, 6, 7, 8

Type material examined.– Mature female holotype (5.7 mm TL), with brood of premolt epicaridium larvae (some mounted on SEM stub) and one cryptoniscus larva of *Bourdonia inexpectata* **n. sp.** in marsupium, from left branchial chamber of male *Gonionida rubrimana* (Ahyong) (8.8. mm CL; det. S. Ahyong), Sta. TAN0107/228, 36.1393333 S, 178.1961667 E to 36.1428333 S, 178.1916667 E [36°08′21.6″S 178°11′46.2″E to 36°08′34.2″S 178°11′30.0″E], Rumble V seamount, off New Zealand, 655–877 m, 24 May 2001, coll. Seamounts of the Outer Bay of Plenty cruise (NIWA 76181). Allotype male (2.2 mm TL), same data as holotype. Mature female paratype (5.4 mm TL) and male paratype (2.1 mm TL) pair, from left branchial chamber of female Munididae sp. (7.6 mm CL, with three mature externae of rhizocephalan *Duplorbis* sp. hyperparasite), Sta. TAN1104/9, 36.504 S, 177.877 E to 36.502 S, 177.873 E [36°30′14.4″S 177°52′37.2″E to 36°30′07.2″S 177°52′22.8″E], Kermadec Ridge, off New Zealand, 1576–1583 m, 3 March 2011, coll. Ocean Survey 20/20-NZASMS (NIWA 160807 ex 85239).

Type locality.– 36.1393333 S, 178.1961667 E to 36.1428333 S, 178.1916667 E [36°08′21.6″S 178°11′46.2″E to 36°08′34.2″S 178°11′30.0″E], Rumble V seamount, off New Zealand, 655–877 m.

Distribution.– Known from the type locality and 36.504 S, 177.877 E to 36.502 S, 177.873 E [36°30′14.4″S 177°52′37.2″E to 36°30′07.2″S 177°52′22.8″E], Kermadec Ridge, off New Zealand, 1576–1583 m.

Host.– Gonionida rubrimana (Ahyong) and Munididae sp. (Anomura: Munididae).

Etymology.– The new species is named in honor of Sadie Mills, collection manager at NIWA, who was incredibly helpful during a research visit by the second author in 2023, facilitated the loan of and supplied information on the specimens.

Description of mature female (Figs. 5B, C, 6).– Holotype female: body length 5.7 mm, maximal width 3.6 mm across pereomeres 3 and 4, head length 1.1 mm, head width 1.3 mm. Body sinuous, ovate, pereon slightly deflexed sinistrally; all body regions and pereomeres distinctly segmented (Figs. 5B, 6A). Head ovate, broader than long, bilobed on posterior margin, frontal lamina broad; eyes absent (Fig. 6A). Barbula with one smooth digitiform lobe on each side bearing scales on distal margin (Fig. 6F inset), median region indented (Fig. 6E, F). Antennules of three articles each, terminal article minute; antennae of four articles each, terminal article approximately half as wide as penultimate article, setose (Fig. 6C). Maxilliped with setae on margin of anterior lobe, palp absent (Fig. 6F); posterior lobe with setose short broadly rounded spur. Oostegite 1 anterior lobe ovate, posterior lobe approximately two times larger than anterior lobe, internal ridge smooth (Fig. 6G, H).

Pereon of seven pereomeres, broadest across pereomeres 3 and 4, gradually tapering anteriorly and posteriorly (Fig. 5B, 6A); pereomere 1 with straight posterior margin; pereomere 2 anteriorly straight, posteriorly weakly concave, pereomeres 3–7 anteriorly convex, posteriorly concave (Fig. 6A). Coxal plates distinct on pereomeres 1–7, recurved on 6 and 7, dorsolateral bosses on pereomeres 1–5, absent on pereomeres 6 and 7, posterior margins smooth (Fig. 6A). Marsupium open (Figs. 5B, 6B). Pereopods subequal, bases without bosses (Fig. 6D, I).

Pleon of six pleomeres including pleotelson; pleomeres 1–5 with rounded lateral plates (Fig. 6A). Pleomeres 1–5 with biramous pleopods, decreasing in size posteriorly, not covering median region of pleon; endopods of 1–4 lanceolate, longer than exopods, pleopod 5 with endopod and exopod rounded, endopod shorter (Fig. 6B); uropods uniramous, each with distal bifurcation, anal cone absent (Fig. 6J).

Allotype male: length 2.25 mm, maximal width 1.0 mm across pereomere 5, head length 0.32 mm, head width 0.60 mm, pleon length 0.48 mm (portion visible in dorsal view). All body segments with numerous spinous scales, most numerous on lateral edges (Fig. 7A inset). Head ovoid, widest midpoint of length, distinct from pereomere 1, medial margin slightly extending one-third distance into pereomere 1; sensory pits present, eyes absent (Fig. 7A). Antennules of three articles each, all articles setose, terminal segment with six setae; antennae of four articles each, all articles setose, terminal segment with six setae (Fig. 7C). Maxilliped two-segmented with three to five terminal thick setae (Fig. 7E).

Pereomeres 4 and 5 broadest, others tapering slightly anteriorly and posteriorly (Fig. 7A). Pereomere 1 posteriorly weakly convex, 2–6 nearly straight, 7 weakly concave, distolateral margins of all pereomeres rounded (Fig 7A); ventral anterolateral edges of pereomeres 3–7 forming extended apophysis, larger on posterior pereomeres (Fig. 7B). Pronounced midventral posteriorly directed tubercle on pereomere 1, ovate midventral tubercles on pereomeres 2–7 (Fig. 7B). Pereopods 1 and 2 each with long, curved dactylus not reaching distal margin of carpus (Fig. 7D), posterior dactyli decreasing in size; propodus large, broad, not fringed with setae on dorsal edge; carpus, small, rounded with distal setose area; merus small, rounded; ischium rounded; basis elongated (Fig. 7D, F).

Pleon of six pleomeres, sides of all pereomeres laterally extended, not overlapping (Fig. 7B); small, rounded pleopods on pleomeres 1–5. Pleotelson curled ventrally, not visible in dorsal view, with articulated, rounded uropods bearing three or four distal setae and extending beyond medially indented anal cone (Fig. 7G).

Description of premolt epicaridium larva (Fig. 8).— Approximately 175 µm in length (anterior margin of head to end of yolk sac). Body tear-drop shaped (Fig. 8B), broadest at anterior end, pereopods extending beyond lateral margin. Segmentation of appendages and body obscured by exoskeleton (future molt). Anterior margin of head rounded, inflated. Antennule short (Fig. 8B, C), with broad basis and evidence of distal setae, antennae long, approximately 3/4 length of body (Fig. 8A), bifid distally at end.

Mouthparts consisting of rounded mandibles with evidence of toothed distal tips between digitate maxillula (= maxilla 1), maxilla (= maxilla 2) longer and thinner than maxillula, maxillipeds nearly same length as maxilla but with bifid tips (Fig. 8D).

Six pairs of gnathopodal pereopods, subequal in size, hooked dactylus and broad propodus visible, rest of articles obscured; pereopods 1–4 oriented laterally, pereopods 5, 6 extending posteriorly among pleopods (Fig. 8A, B, E).

Pleon with five pairs of biramous pleopods (Fig. 8F, G), articles obscured but exopods appear to have three stout setae. Uropods biramous, distal ends bifid (Fig. 8F–H). Ovate to round yolk sac (Fig. 8A, F–H), extending to base between uropods, margin of yolk sac with ridge in cuticle (Fig. 8A, F, G).

Remarks.– *Paragigantione sadieae* **n. sp.** is most similar to *P*. *papillosa* and *P*. *indica* but can be distinguished from those species as follows: 1) female body shape of *P*. *sadieae* **n. sp.** with pronounced “shoulder” (expanded pereomeres 2–4 on long side of body, also seen in *P*. *indica*) vs. body nearly straight in *P*. *papillosa*, 2) antennae of four articles each in *P*. *sadieae* **n. sp.** vs. five articles each in *P*. *papillosa* and six articles each in *P*. *indica*, 3) oostegite 1 of *P*. *sadieae* **n. sp.** with anterior and posterior lobes subequal in length vs. oostegite 1 of the other two species with the posterior lobe much longer than the anterior lobe and 4) male of *P*. *sadieae* **n. sp.** with pleotelson not extending beyond distal margin of pleotelson and with pleotelson not visible in dorsal view vs. *P*. *indica* with pleotelson not extending beyond distal margin of pleotelson but with pleotelson visible in dorsal view and *P*. *papillosa* with pleotelson extending beyond distal margin of pleotelson and pleotelson visible in dorsal view. As epicaridium larvae of *P*. *papillosa* and *P*. *indica* are unknown, no comparison of this life history stage with that of *P*. *sadieae* **n. sp.** is possible.

**Key to females of species of** ***Paragigantione*** **Barnard, 1920**

1a Some tergal projections with crenulate posterior margins ……… 2

1b All tergal projections with smooth posterior margins ……… 3

2a Pleopod 1 endopod 1.3x as long as exopod ……… *P*. *americana* (Markham, 1974b)

2b Pleopod 1 endopod 3.5x as long as exopod ……… *P*. *europaea* **n. sp.**

3a Oostegite 1 with anterior and posterior lobes subequal in length ……… *P*. *sadieae* **n. sp.**

3b Oostegite 1 with posterior lobe much longer than anterior lobe ……… 4

4a Uropods bifurcated approximately ½ length ……… *P*. *indica* Nierstrasz & Brender à Brandis, 1923

4b Uropods bifurcated nearly entire length ……… *P*. *papillosa* Barnard, 1920

**Key to males of species of** ***Paragigantione*** **Barnard, 1920**

1a Midventral tubercle on pleomeres 1 and 2 ………*P*. *americana* (Markham, 1974b)

1b No midventral tubercle on pleomeres ……… 2

2a Midventral tubercles on pereomeres 1–7……… *P*. *europaea* **n. sp.**

2b Midventral tubercles on pereomeres 1–6……… 3

3a Posterolateral lobes of pleomere 5 extending beyond distal margin of pleotelson……… *P*. *papillosa* Barnard, 1920

3b Posterolateral lobes of pleomere 5 not extending beyond distal margin of pleotelson.……… 4

4a Pleotelson visible in dorsal view ……… *P*. *indica* Nierstrasz & Brender à Brandis, 1923

4b Pleotelson not visible in dorsal view……… *P*. *sadiae* **n. sp.**

Superfamily Cryptoniscoidea Kossmann, 1880

Family Cabiropidae Giard & Bonnier, 1887

*Bourdonia* Rybakov, 1990

Type species.— *Bourdonia tridentata* Rybakov, 1990, by monotypy.

**Fig. 9:**
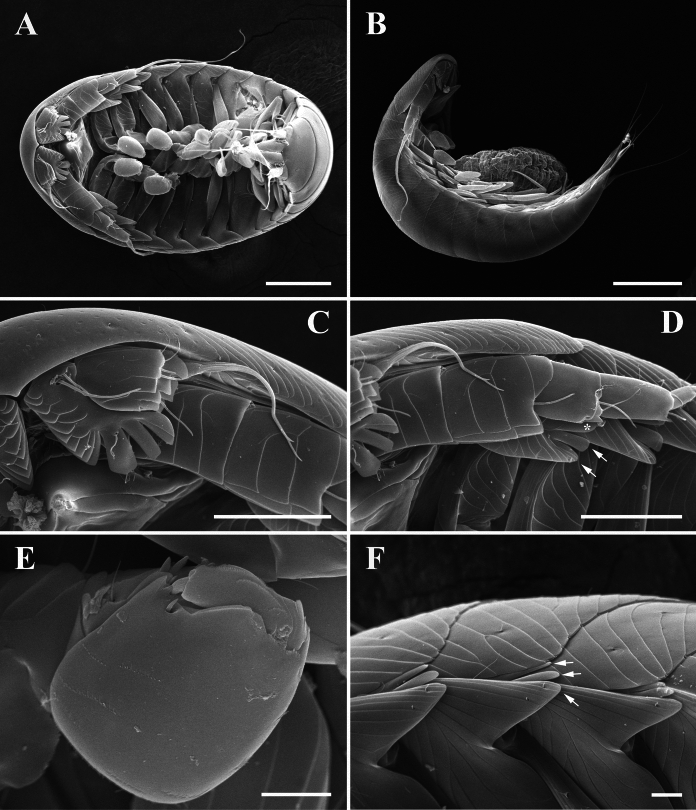
Holotype cryptoniscus larva of *Bourdonia inexpectata* **n. sp.**, hyperparasite of female *Paragigantione sadieae* **n. sp.** **A**, Ventral view; **B**, Lateral view; **C**, Left antennule; **D**, Penduncular segments of antennae and coxal plates 1 and 2, teeth shown with arrows on coxal plate 1 (asterisk shows edge of pereon that could be confused with a third tooth); **E**, Pereopod 2; **F**, Coxal plates 5–7, teeth shown with arrows on coxal plate. *Scale-bars*: **A**, **B**, 100 µm, **C**, **D**, 50 µm, **E**, **F**, 10 µm.

**Fig. 10:**
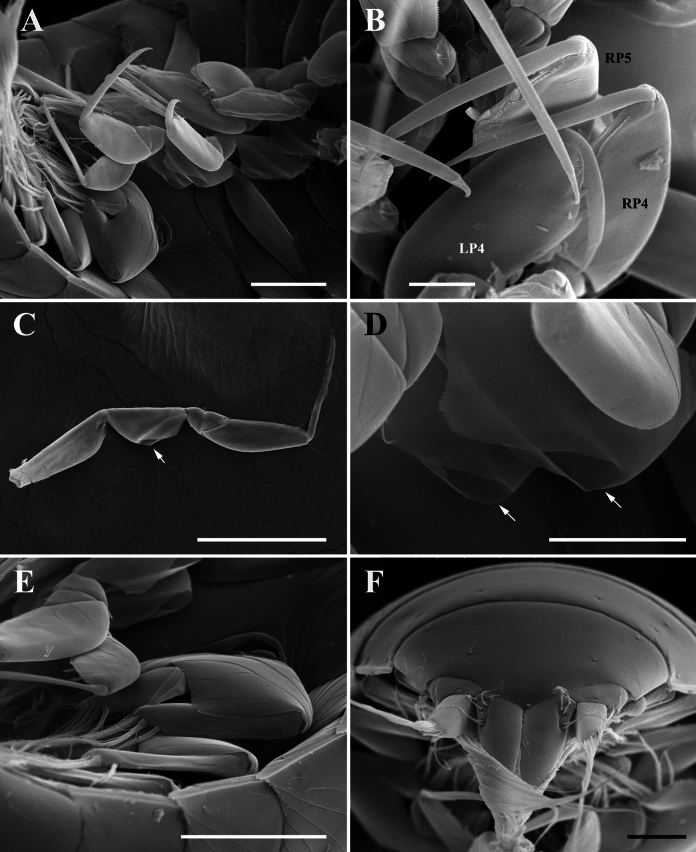
Holotype cryptoniscus larva of *Bourdonia inexpectata* **n. sp.**, hyperparasite of female *Paragigantione sadieae* **n. sp.** **A**, Ventral view showing pereopods 2–7 and pleopods; **B**, Pereopods 4–6; **C**, Left pereopod 7, groove in ischium shown by arrow; **D**, Ischium of pereopods 6 and 7, arrows showing groove; **E**, First pleopod; **F**, Pleon, dorsal view. Abbreviations: Abbreviations: LP4 = left pereopod 4; RP4, RP5 = right pereopods 4, 5. *Scale-bars*: **A**, 50 µm, **B**, 15 µm, **C**, 100 µm, **D**, 20 µm, **E**, 50 µm, **F**, 25 µm

**Fig. 11:**
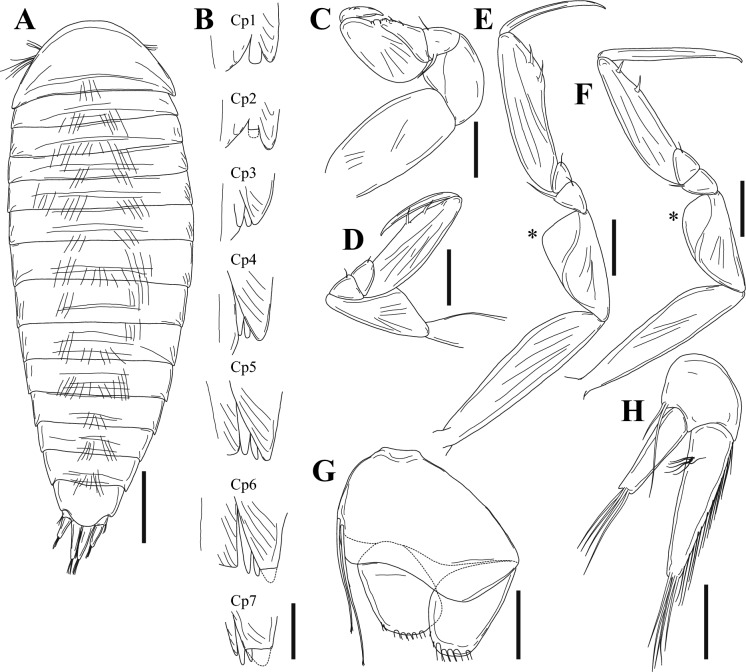
Paratype cryptoniscus larva of *Bourdonia inexpectata* **n. sp.**, hyperparasite of female *Pseudione* cf. *fibriata* Richardson, 1910. **A**, Dorsal view; **B**, Coxal plates 1–7 showing teeth, dashed lines indicate broken portions; **C**, Left pereopod 2; **D**, Left pereopod 3; **E**, Left pereopod 4, asterisk shows position of groove in ischium; **F**, Left pereopod 7, asterisk shows position of groove in ischium; **G**, Left pleopod 1, insertion points of setae on endopod and exopod shown. **H**, Left uropod. *Scale-bars*: **A**, 200 µm, **B–H**, 50 µm

***Bourdonia inexpectata*** **n. sp.** Figs. 9, 10, 11

Type material examined.– Holotype cryptoniscus larva (950 µm TL, ex *Paragigantione sadieae* **n. sp.** from left branchial chamber of male *Gonionida rubrimana* (Ahyong) (Munididae) (8.8 mm CL; det. S. Ahyong), Sta. TAN0107/228, 36.1393333 S, 178.1961667 E to 36.1428333 S, 178.1916667 E [36°08′21.6″S 178°11′46.2″E to 36°08′34.2″S 178°11′30.0″E], Rumble V seamount, off New Zealand, 655–877 m, 24 May 2001, coll. Seamounts of the Outer Bay of Plenty cruise (NIWA 173609 ex 76181). Paratype cryptoniscus larva (1.5 mm TL, ex *Pseudione* cf. *fibriata* Richardson, 1910 (Pseudioninae) from right branchial chamber of female *Munidopsis victoriae* Baba & Poore (Munidopsidae) (15.0 mm CL; det. K. Schnabel), Sta. TAN0509/43, 42.8416672 S, 178.9246674 E to 42.8428345 S, 178.9586639 E [42°50′30.0″S 178°55′28.8″E to 42°50′34.2″S 178°57′31.2″E], off New Zealand, 1004–1007 m, 28 June 2005, coll. Ministry of Fisheries, NIWA (NIWA 173610 ex 19231B).

Type locality.– 36.1393333 S, 178.1961667 E to 36.1428333 S, 178.1916667 E [36°08′21.6″S 178°11′46.2″E to 36°08′34.2″S 178°11′30.0″E], Rumble V seamount, off New Zealand, 655–877 m.

Distribution.– Known from the type locality and 36.1393333 S, 178.1961667 E to 42.8428345 S, 178.9586639 E [42°50′30.0″S 178°55′28.8″E to 42°50′34.2″S 178°57′31.2″E], off New Zealand, 655-1007 m.

Hosts.– *Paragigantione sadieae* **n. sp.** (Bopyridae: Pleurocryptellinae) and *Pseudione* cf. *fibriata* Richardson, 1910 (Bopyridae: Pseudioninae).

Etymology.– Rybakov (1990) did not specify the gender of *Bourdonia* but the type species is *Bourdonia tridentata*, which means *Bourdonia* is feminine. The new species name is given because the finding of this cryptoniscus was unexpected (see Remarks).

Description of cryptoniscus larva (Figs. 9, 10, 11).— Body cylindrical, elongated (Figs. 9A, B, 11A), holotype length 950 µm, widest at pereomeres 4, 5 (~300 µm), anterior and posterior pereomeres narrower. Head anterior margin round, medial region of posterior margin approximately straight on dorsal side, posterolateral margins extending posteriorly, partially overlapping pereomere 1, anterior margin of head on ventral side closely applied to antennules, with short medial extension between antennules (Fig. 9A, C). Cuticle striated on dorsal surface of pereomeres with few scattered setae, striations also on antennae and pereopods (Figs. 9B–D, F, 11A, B).

Antennules of three articles each (Fig. 9A, C), basal article with four large teeth on posterior margin, anteromedial corner with three large setae and few smaller setae toward margin, article 2 quadrate with large distal teeth and scales, three large setae and two smaller setae on anteromedial corner, article 3 small, lobe-like with at least two long distal setae, an additional lobe is present with multiple long setae but obscured by head. Antennae of nine articles each (four peduncular, five flagellar) (Fig. 9A, B, D), articles 1–4 tubular with distal setae, article 2 largest with apophysis, article 3 with two plumose setae and one stout seta, flagellar articles much smaller in diameter than distal peduncular article, flagellar articles progressively longer and with terminal setae, two long setae on distal article. Oral cone triangular, anteriorly directed (Fig. 9A–C).

Pereomeres 1–7 with toothed coxal plates, pereomeres 1 and 2 with two coxal teeth (Figs. 9D, 11B), pereomeres 3–7 with three coxal teeth each (Figs. 9F, 11B) (see Remarks on counts of coxal teeth). Pereopods 1 and 2 gnathopodal (Figs. 9A, E, 11C), each with short curved dactylus bearing spinous scales on top and pointed tip, propodus semi-spherical with small setae on side of dactylus. Pereopods 3–5 (Figs. 9A, 10A, B, 11D, E) with dactylus slender, elongated, extending beyond expanded distal end of propodus, propodus expanded distally with short cutting edge lined with spinous scales and two club-like thick spines; carpus small, triangular with spine; ischium broad, triangular with “shelf” (see Remarks) in form of a groove with setules along the outer ridge (Figs. 10A, 11E, F). Pereopods 6 and 7 (Figs. 9A, 10A, C, 11F) with dactylus slender, elongated, extending beyond expanded distal end of propodus, propodus tubular with long cutting edge lined with spinous scales, two stout setae toward distal end, on side of dactylus; carpus small, triangular with spine; ischium broad, triangular with groove (see Remarks) bearing setules along the outer ridge (Figs. 9C, D, 11F).

Pleon with five pairs of biramous pleopods (Figs. 10E, 11G), each sympod broad, quadrate, distal margin with thin extension of cuticle overlapping basal portion of endopod and exopod, sympod with two long lateral setae with multifid tips (Fig. 11G); endopods and exopods plate-like; endopod and exopod of pleopod 1 each bearing four long plumose setae and one shorter lateral plumose seta (Fig. 10E); endopod and exopod of pleopod 5 each bearing three long plumose setae and one shorter lateral plumose seta. Pleotelson (Figs. 10F, 11A) with distomedial rounded projection, concave on each side. Uropods biramous (Figs. 10F, 11H), each composed of subquadrate sympod with six setae on distal corners, endopod cylindrical, tapering, with curled setae toward base, numerous shorter setae along medial edge, few long distal setae; exopod cylindrical, tapering, approximately 1/2 length of endopod, with long simple setae.

Remarks.— The finding of this hyperparasite was unexpected; the first specimen was found while preparing the epicaridium larvae of *Paragigantione sadieae* **n. sp.** for SEM examination and after the larvae had been coated and placed on an SEM stub. A second specimen was also unexpectedly discovered parasitizing a bopyrid belonging to a different subfamily extracted from a squat lobster of a different family than the one from which the holotype was obtained. The hyperparasite has the typical cryptoniscoid antennae segment count (four basal and five flagellar segments) and pereopods 1 and 2 are gnathopodal but without the bifid tips found in cryptoniscus larvae of all species of *Cabirops* Kossmann, 1884.

We place this species in *Bourdonia* Rybakov, 1990 as the cryptoniscus larvae share two key characters with the type and previously only known species in that genus, *B*. *tridentata* Rybakov, 1990: a strongly toothed antennular basal segment and lack of bifid tips on the dactyli of the anterior pereopods. The shape of the pleotelson and the presence of two spines on the cutting edge of the propodi of pereopods 3–5 are also seen in species of *Cabirops* but those species have antennular basal segments without teeth and have bifid tips on the anterior pereopodal dactyli. One other species in a monotypic genus shows some similarities to the new species: *Rolandoniscus serratus* (Bourdon, 1967), whose cryptoniscus larvae also lack bifid tips on the anterior pereopods and have teeth on the antennal basal segment but have three spines on the cutting edge of the propodi of pereopods 3–5, a very differently shaped pleotelson, and an unusual coxal plate dentition formula of 4:4:1:1:1:1:1 (see Bourdon, 1967). The coxal plate formula of *B*. *tridentata* is 3:3:3:3:3:3:2 whereas in the new species it is 2:2:3:3:3:3:3. We believe that the coxal plate formulae have been counted misleadingly by other authors, due to the fact that the innermost “tooth” is part of the pereopodal coxa (as evidence by the presence of cuticular striations on its surface; see Figs. 9D, E, 11B); however, we maintain the traditional method of counting (e.g., Sassaman, 1985) for the present to avoid confusion. This is the first record of a hyperparasite from any species of *Paragigantione*; the host of the paratype may be *Pseudione fibriata* or a new species that is closely related to it. The presumptive new species of rhizocephalan from the genus *Duplorbis* Smith, 1906 is also a hyperparasite (see Mourey, 1991 and Høeg et al., 2020 for information on these poorly known parasitic barnacles) and will be described in a future paper (Williams et al., in prep); this is the first record of any rhizocephalan parasitizing a species of *Paragigantione*.

A note on the structure of the ischium of cryptoniscus larvae is warranted. Many authors (e.g., Bourdon, 1967; Williams et al., 2024) have noted, either in description or figures, the presence of a large, thin “shelf” or blade on the upper edge of the pereopodal ischia. This is not a solid structure or simple thin cuticular extension as had been assumed by many prior researchers, it is actually an extension that forms a groove into which the merus articulates, as noted by Hosie (2008). It is likely that many, if not all, epicaridean cryptoniscus larvae have this type of mechanism, the function of which may be to keep the pereopods adpressed to the body, and therefore reduce drag, when the cryptoniscus is moving through the water column via propulsion of the pleopods.

## Discussion

The present study shows that *Pleurocryptella* and *Paragigantione* belong in the same subfamily, Pleurocryptellinae, based on the discovery of posterior, external yolk sacs on the epicaridium larvae in two species of *Paragigantione* that are also found in those of *Pleurocryptella* and are presently known from no other genus. Species in both genera also have epicaridium larvae and males that retain segmented maxillipeds, female with rounded anterior and posterior segments of oostegite 1, and males that possess uropods; these characters taken together distinguish the two genera from all those belonging to Pseudioninae. Unfortunately, there is no molecular data available for *Paragigantione*, but *Pleurocryptella* 18S rRNA and COI data has shown it to be distinct from Pseudioninae (Kato et al., 2022; Williams et al., 2024). Further studies on males and females of species in genera such as *Gigantione* Kossmann, 1881, *Parapleurocryptella* Bourdon, C, and *Pagurocryptella* Boyko & Williams, 2010 that have some shared and unusual character states with *Pleurocryptella* and *Paragigantione* are needed, ideally supported by larval and molecular data that are lacking for all species in these genera. The discovery of another new species of hyperparasite in the marsupium of a female of *Paragigantione sadiae* **n. sp.** shows that the diversity of these epicarideans is greatly underestimated.

## Data Availability

Type material, and all specimens examined, are deposited in the Voss Marine Invertebrate Collections, Rosenstiel School of Marine and Atmospheric Science, University of Miami (UMML) and the National Institute of Water & Atmospheric Research Ltd (NIWA), Wellington, New Zealand (see text for details) and are available for study.
